# Lasting Effects on Body Weight and Mammary Gland Gene Expression in Female Mice upon Early Life Exposure to *n*-3 but Not *n*-6 High-Fat Diets

**DOI:** 10.1371/journal.pone.0055603

**Published:** 2013-02-07

**Authors:** Mirjam Luijten, Amar V. Singh, Caleb A. Bastian, Anja Westerman, M. Michele Pisano, Jeroen L. A. Pennings, Aart Verhoef, Maia L. Green, Aldert H. Piersma, Annemieke de Vries, Thomas B. Knudsen

**Affiliations:** 1 Laboratory for Health Protection Research, National Institute for Public Health and the Environment, Bilthoven, The Netherlands; 2 Department of Molecular, Cellular and Craniofacial Biology, University of Louisville Birth Defects Center, Louisville, Kentucky, United States of America; 3 National Center for Computational Toxicology, U.S. Environmental Protection Agency, Research Triangle Park, North Carolina, United States of America; Wayne State University School of Medicine, United States of America

## Abstract

Exposure to an imbalance of nutrients prior to conception and during critical developmental periods can have lasting consequences on physiological processes resulting in chronic diseases later in life. Developmental programming has been shown to involve structural and functional changes in important tissues. The aim of the present study was to investigate whether early life diet has a programming effect on the mammary gland. Wild-type mice were exposed from 2 weeks prior to conception to 6 weeks of age to a regular low-fat diet, or to high-fat diets based on either corn oil or flaxseed oil. At 6 weeks of age, all mice were shifted to the regular low-fat diet until termination at 10 weeks of age. Early life exposure to a high-fat diet, either high in *n*-6 (corn oil) or in *n*-3 (flaxseed oil) polyunsaturated fatty acids, did not affect birth weight, but resulted in an increased body weight at 10 weeks of age. Transcriptome analyses of the fourth abdominal mammary gland revealed differentially expressed genes between the different treatment groups. Exposure to high-fat diet based on flaxseed oil, but not on corn oil, resulted in regulation of pathways involved in energy metabolism, immune response and inflammation. Our findings suggest that diet during early life indeed has a lasting effect on the mammary gland and significantly influences postnatal body weight gain, metabolic status, and signaling networks in the mammary gland of female offspring.

## Introduction

Over the past decade, numerous epidemiological studies have shown strong inverse associations between birth weight and risk of cardiovascular disease, hypertension, type 2 diabetes, and other diseases in adult life [1]–[4]. These findings have been largely supported by the results of studies in experimental animals [5], [6]. Barker and colleagues originally put forward the hypothesis that poor fetal growth results in an increased risk for coronary heart disease [7], [8]. The fetal origins of adult disease hypothesis or ‘Barker hypothesis’ states that adverse environmental factors during fetal life, particularly nutrition, ‘program’ the individual for an increased risk of major chronic diseases [9]–[13].

Studies on the Barker hypothesis have mainly focused on the impact of maternal undernutrition. However, in developed countries maternal and postnatal nutrition are now either sufficient or excessive. Not only maternal undernutrition, but also an imbalance of nutrition in the opposite direction has been demonstrated to correlate with adult diseases, such as type 2 diabetes and obesity [14]–[17]. Breast cancer incidence rates are about four-fold higher in Western Europe and North America than in Asia and Africa [18], [19]. Migrants from low- to high-risk countries acquire breast cancer rates of their adopted country within two generations [20]. Diet is, among other lifestyle factors, one of the major risk factors. It is well established that high levels of dietary fat increase the development of mammary tumors in rodents, although it is dependent upon period of exposure and types of dietary fat [21]–[25]. Consumption of a high-fat diet has also been shown to increase breast cancer risk in humans [26]–[29]. Breast cancer in particular was identified as a disease with a dominant link to birth weight: individuals with higher birth weight have an increased risk of developing breast cancer [23], [30]–[32]. The underlying mechanisms still need to be elucidated, but these may involve changes in epigenetic processes leading to altered gene activity and hence cellular dysregulation [30], [31].

Developmental programming involves structural and functional changes in important tissues leading to altered cell number, imbalance in distribution of different cell types within the organ, and altered blood supply or receptor numbers [5]. Tissues and organs that have been identified as targets for programming include heart, kidney, liver, pancreas, and adipose tissue [33]. Differentiation of the mammary gland has also been demonstrated to be sensitive to changes in diet during early life [34], [35]. Prenatal and postnatal dietary changes influence further development of the mammary gland and, ultimately, might induce persistent morphological changes in the mammary gland that in turn modify breast cancer risk later in life. Studies in rodents have, in general, be supportive of a tumor-promoting effect of consumption of a diet rich in *n-*6 polyunsaturated fatty acids (PUFAs) during early life, whereas consumption of a diet with a lower *n*-6/*n*-3 PUFA ratio is potentially associated with a decreased incidence of cancer [23], [24], [36]–[39].

The goal of the present study was to determine the extent to which diet in early life has a lasting effect on mammary gland development and to identify the underlying cellular-response pathways based on genome-wide analysis of the transcriptome. We investigated the effect of different types of dietary fat, using high-fat diets high in *n*-6 or in *n*-3 PUFAs, and studied gene expression profiles in relation to postnatal growth curves in mice. Mice were exposed to different experimental diets through the mother, starting 2-weeks pre-conception and continuing prenatally through *in utero* and lactational periods, then from weaning (3 weeks) until the age of 6 weeks, before switching them to standard mouse chow. Final assessments were done in sexually mature nulliparous females at 10 weeks of age. This study design does not take into consideration the further development of the mammary gland during pregnancy and lactation. Rather, our question focused on whether at this stage, with puberty and adolescence completed, treatment-related differences in mammary gland development could be observed that might predispose the mammary gland to pathogenesis at later life-stages. Later life-stages could of course include changes triggered by pregnancy and lactation.

## Results

**Figure 1:**
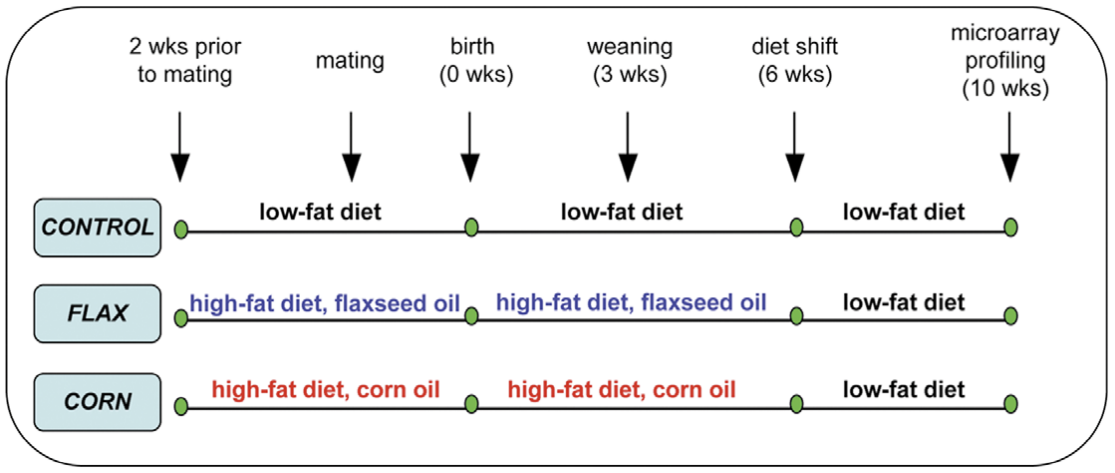
Schematic overview of experimental study design. Wild-type FVB mice were exposed from two weeks prior to conception to 6 weeks of age to a low-fat (5%) regular mouse diet, or to high-fat (24%) diets based on either corn oil or flaxseed oil. At 6 weeks of age, all mice were shifted to the regular low-fat diet until terminal sacrifice at 10 weeks of age.

To determine whether nutrition in early life has a lasting effect on the mammary gland and to obtain insight in the underlying pathways, we fed wild-type mice from 2 weeks prior to conception to 6 weeks of age either a low-fat diet or a high-fat diet based on corn oil (CO, high in *n*-6 PUFAs) or flaxseed oil (FO, high in *n*-3 PUFAs). In line with published literature [40], [41] we envisaged that 2 weeks would be sufficient for the animals to adapt their physiology to the diet before pregnancy is initiated. Preliminary findings showed that continuous exposure to a high-fat diet (e.g., pups not shifted to low-fat diet at 6 weeks of age) caused the mice to become obese (≥40 g) by 35 weeks of age. To bring the study to a more ethical standard we shortened the exposure to high-fat diet by putting dams on the high-fat diet from 2 weeks pre-conception to lactation and then placing the female offspring on the high-fat diet for 3 weeks post-weaning, before switching them to a regular low-fat diet. This meant females received the test diets through 6 weeks of age. At 10 weeks of age, mice were euthanized and gene expression profiling of the abdominal mammary glands was performed. A schematic overview of the study design is depicted in Figure 1.

### Early Life Exposure to High-fat Diets did not Affect Fertility

Early life dietary exposure to a high-fat diet, rich in either *n*-6 or *n*-3 PUFAs, did not result in significant effects on pregnancy rate, litter size or sex ratio (Table 1). There was no evidence of filial cannibalism. The number of female pups born in each dietary group was *n* = 21 for the control group, *n* = 12 for the corn oil (CO) group, and *n* = 15 for the flaxseed oil (FO) group.

**Table 1:** Murine Offspring at Parturition.

Parameter	Low-fat(control)	High-fat(corn oil)	High-fat (flaxseed oil)
Number of litters*	5 *(6)*	4 *(6)*	5 *(6)*
Average litter size	8±1.58	8±0.82	7.4±1.52
Total live-born pups	40	32	37
Sex ratio (male/female)	0.90	1.67	1.47
Total # female pups	21	12	15
Total # male pups	19	20	22

* The total number of F0 female mice in the study is indicated between parentheses.

### Changes in Serum Fatty Acid Profile

To investigate whether perinatal exposure to treatment diets high in *n*-6 or *n*-3 PUFAs was still detectable at time of sacrifice (*i.e.* 4 weeks after cessation of treatment), fatty acid profiling of a representative number of mouse serum samples was performed. Results are summarized in Table 2. Sera of mice from the FO diet group still reflected treatment to a diet high in *n*-3 PUFAs. Mice fed the FO diet had over twofold higher serum levels of *n*-3 PUFAs α-linolenic acid, eicosapentaenoic acid, and docosahexaenoic acid (*P*<0.001), and about twofold lower serum levels of the *n*-6 PUFA arachidonic acid (*P*<0.001), compared to mice fed either the control or the CO diet. The levels of linoleic acid were significantly increased in the high-fat diet groups compared to the control group (*P*<0.01), although to a limited extent. Overall, the *n*-6 to *n*-3 PUFA ratio was about threefold lower in mice fed the FO diet as compared with mice fed the CO diet.

**Table 2:** Fatty acid profile of mouse sera†.

Fatty acids		Low-fat (control)		High-fat (corn oil)		High-fat (flaxseed oil)	
		Mean‡	SD	Mean	SD	Mean	SD
Saturated FA		28.84^a^	0.59	29.14^a^	0.73	29.12^a^	0.57
*n*-3 PUFA	ALA	0.77^a^	0.084	0.84^a^	0.10	2.59^b^	0.62
	EPA	0.27^a^	0.052	0.23^a^	0.11	2.11^b^	0.52
	DHA	2.99^a^	0.23	2.84^a^	0.25	4.11^b^	0.40
	total	4.25^a^	0.24	4.14^a^	0.38	9.55^b^	1.25
*n*-6 PUFA	LA	23.76^a^	1.27	26.20^b^	0.51	28.47^c^	1.57
	AA	14.52^a^	1.46	15.41^a^	1.12	7.57^b^	1.34
	total	39.68^a^	2.14	43.12^b^	0.78	37.37^c^	2.43
*n*-7cis PUFA		4.25^a^	0.93	3.33^b^	0.65	3.24^b^	0.34
*n*-9cis PUFA		17.22^a^	1.72	15.46^b^	0.94	15.56^b^	1.44
*n*-6 to *n*-3 ratio		9.34		10.42		3.91	

† Serum analyses: *n* = 8−10 per group; animals were not fasted prior to measurement.

‡ Percent of total fatty acids.

a,b,c Mean values within a row with unlike superscript letters were significantly different (ANOVA; *P<*0.05).

### Postnatal Body Weight Gain

**Figure 2:**
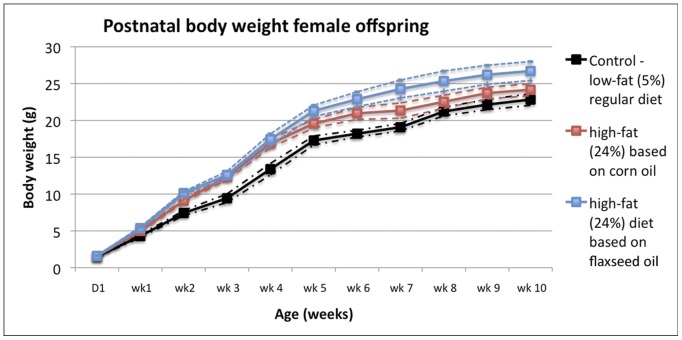
Body weight trajectories in F1 female mice, perinatally exposed to a low-fat (5%) regular mouse diet (indicated in black), or to high-fat (24%) diets based on either corn oil (red) or flaxseed oil (blue). Graph plots mean (and 95% confidence intervals) body weights of F1 female pups with age. The increased body weights of mice fed high-fat diets were significant versus regular diet at 3 weeks of age and beyond (*P*<0.001; nonlinear repeated measures model); however, mice fed the high-fat corn oil diet shifted to the regular trajectory between 6 and 10 weeks of age. The slight dip in the growth curves at 3 weeks of age in all three groups is accounted for by the removal of male pups, which are slightly heavier than females.

Body weights of the F1 mice were recorded at the day of birth (PND 0) and regular intervals thereafter. The resulting growth curves are shown in Figure 2. No significant group differences in pup body weight was observed on PND 1; however, female pups that had been exposed prenatally to a high-fat diet were significantly heavier than their counterparts fed the low-fat diet by 3 weeks of age due to more rapid weight gain (*P*<0.001). A slight dip in the growth curves at 3 weeks of age in all three groups was accounted for by the removal of male pups, which are slightly heavier than females. Subsequent body weight trajectories of the female pups varied between FO and CO groups. Postnatal growth curves of female pups sustained on the FO diet generally paralleled the normal growth curve through 10 weeks of age, but remained heavier by approximately 4–5 grams. In contrast, the growth curve of female pups sustained on the CO diet gradually approached the curve of the control group after 4 weeks of age. At 10 weeks of age, body weights of the F1 female pups from the CO diet group were no longer significantly different from the control group whereas female pups that were fed the FO diet remained significantly heavier than pups from the control group (*P*<0.001) despite being shifted to a low-fat diet from 6 weeks of age onwards. The relative mass of the mesenteric adipose tissue mass was not significantly different between groups (data not shown). These results on body weight gain show a more persistent effect for females exposed to the FO diet than the CO diet and furthermore suggests that both the nature and degree of dietary fat exposure in early life is an important parameter in this animal model.

### Maternal Dietary Fat Exposure Influences Developmental Programming

**Figure 3:**
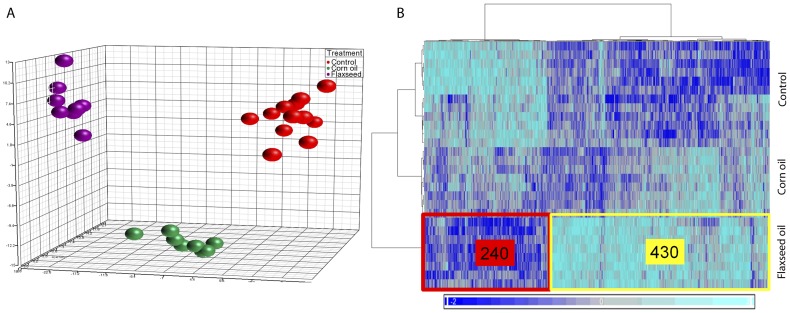
Gene expression changes in the mammary gland. Test diets were given from two weeks prior to conception to 6 weeks of age, and regular diet thereafter. Microarray analysis was performed at 10 weeks of age. After removing litter-size and batch effects the source of variance was plotted for regular diet (red; *n* = 12), high-fat diet based on corn oil (green, *n* = 8) and high-fat diet based on flaxseed oil (magenta, *n* = 8) using PCA. The heat-map shows clustering of 670 differentially-regulated genes (horizontal) and samples (vertical).

Gene expression of the 4^th^ right abdominal mammary gland of female pups was profiled at postnatal week 10. Differences in gene expression between the groups were quantified using one-way ANOVA and a significance level of *P*<0.005. This analysis revealed 670 genes that accurately classified samples by treatment (Supplemental Table S1). Principal components analysis showed clear evidence of clustering of samples by diet group (Figure 3A). Class differences were greatest between the control and the FO groups, with the CO group being intermediate in its response. This pattern was similar to that observed for body growth curves (Figure 2). Differential transcript abundance profiles could be partitioned neatly into two clusters of genes: 240 genes that were down-regulated, and 430 genes that were up-regulated by FO diet compared to the control group, and to a lesser degree the CO group (Figure 3B).

To gain more insight into the observed changes in gene expression, we first focused on the comparison FO versus control samples. Using the GeneGo MetaCore platform and a stringent significance level of FDR<0.005, we identified over 150 GO biological processes that were significantly over-represented from among the differentially regulated genes identified across the mammary gland samples. Subsequently, pathway analyses were performed using two different approaches. To assign the differentially expressed genes to functional pathways the GeneGo MetaCore Pathway Analysis software was used. In addition, whole data sets were analyzed using GSEA to identify groupwise regulation of GO categories and KEGG pathways. In Metacore, 13 pathways (Table 3) were significantly changed (FDR<0.05). Six of these pathways reflect inflammatory responses, such as interleukin-2 (IL-2), IL-15, and toll-like receptor- (TLR-) signaling. Several key genes of these pathways were significantly up-regulated in the FO group compared to controls, including the IL-2 receptor gamma chain (*Il2rg*), Janus kinase-1 (*Jak1*), *Stat6*, tyrosine kinases *Syk* and *Lck*, and toll-like receptors *Tlr4*, *Tlr6*, and *Tlr8*. Up-regulation of *Syk* as well as *Il4* and chemokine ligand 3 (*Ccl3)*, two other genes involved in inflammation, was confirmed by real-time quantitative PCR (qRT-PCR; Table 4). Two other functional classes were energy metabolism (oxidative phosphorylation, citrate cycle, ubiquinone metabolism, and gluconeogenesis/glycolysis), and cell adhesion (extracellular matrix remodeling). The marked down-regulation of oxidative phosphorylation was confirmed by qRT-PCR analysis of two cytochrome c oxidase subunits: *Cox7a1* and *Cox8b* (see Table 4). Using GSEA (*P*<0.05; FDR<0.1) we found in total 40 enriched pathways (Table 5), all down-regulated by FO treatment and mostly involved in energy metabolism. Examples are oxidative phosphorylation, citrate cycle, fatty acid metabolism, oxidoreductase activity, and electron carrier activity. In contrast to the FO group, a comparison between CO and control groups revealed only 68 differentially expressed genes and among these genes no over-represented GO biological processes or pathways could be identified using MetaCore.

**Table 3:** GeneGo pathway maps over-represented in genes differentially expressed in flaxseed oil-fed mice compared to controls.

GeneGo Pathway Maps	*P*-value (FDR<0.05)	Count*
Oxidative phosphorylation	9.691E-11	22/105
Citric acid cycle	3.888E-6	11/51
Immune response_MIF - the neuroendocrine-macrophage connector	1.000E-5	10/46
Development_Transcription regulation of granulocyte development	2.660E-5	8/32
Immune response_Antigen presentation by MHC class II	6.135E-5	5/12
Immune response_IL-2 activation and signaling pathway	1.141E-4	9/49
Atherosclerosis_Role of ZNF202 in regulation of expression of genes involved in Atherosclerosis	1.2519E-4	6/21
Ubiquinone metabolism	1.503E-4	11/74
Immune response_IL-15 signaling via JAK-STAT cascade	2.175E-4	6/23
Glycolysis and gluconeogenesis	2.893E-4	10/67
Immune response_TLR signaling pathways	3.292E-4	9/56
Cell adhesion_PLAU signaling;	7.667E-4	7/39
Cell adhesion_ECM remodeling	9.500E-4	8/52

* Ratio of number of regulated genes over total number of genes in pathway.

**Table 4:** Expression levels of selected genes in the CO and FO groups.

Gene	Genesymbol	Microarray		qRT-PCR	
		CO	FO	CO	FO
Chemokine (C-C motif)ligand 3	*Ccl3*	0.97*	2.76	1.30	30.96
Cytochrome c oxidasesubunit VIIa 1	*Cox7a1*	0.71	0.53	0.65	0.43
Cytochrome c oxidasesubunit VIIIb	*Cox8b*	0.77	0.64	0.65	0.49
Interleukin 4	*Il4*	1.14	1.45	0.77	2.40
Spleen tyrosine kinase	*Syk*	1.11	1.37	1.04	1.80

* Values indicate fold change in expression level compared to control samples.

**Table 5:** GSEA pathways down-regulated due to treatment with FO diet, as compared to control samples.

Pathway	Nominal*P*-value*	FDRq value	Pathway (*continued*)	Nominal*P*-value	FDRq value
Organelle inner membrane	0.0000	0.0185	Cellular lipid catabolic process	0.0088	0.0519
Microbody	0.0000	0.0179	Mitochondrial membrane	0.0100	0.0163
Peroxisome	0.0000	0.0161	Cofactor metabolic process	0.0101	0.0600
Transferase activity – transferring acyl groups	0.0000	0.0172	Hsa00620: Pyruvate metabolism	0.0105	0.0581
Transferase activity – transferring groups other thanamino acyl groups	0.0000	0.0214	Detection of stimulus involved in sensory perception	0.0122	0.0668
Mitochondrial envelope	0.0020	0.0142	Oxidoreductase activity	0.0123	0.0616
Mitochondrion	0.0021	0.0132	Energy derivation by oxidation of organic compounds	0.0125	0.0533
Hsa00020: Citrate cycle	0.0021	0.0433	Hsa00071: Fatty acid metabolism	0.0126	0.0468
Electron carrier activity	0.0040	0.0173	Lipid catabolic process	0.0128	0.0592
Coenzyme metabolic process	0.0041	0.0508	Mitochondrial respiratory chain	0.0166	0.0221
Mitochondrial inner membrane	0.0041	0.0182	Aerobic respiration	0.0189	0.0585
Cellular respiration	0.0042	0.0435	Hsa00030: Pentose phosphate pathway	0.0199	0.0725
Envelope	0.0058	0.0584	Mitochondrion organization and biogenesis	0.0218	0.0795
Organelle envelope	0.0058	0.0563	Mitochondrial lumen	0.0219	0.0469
Mitochondrial part	0.0059	0.0175	Mitochondrial matrix	0.0219	0.0445
Mitochondrial membrane part	0.0060	0.0117	Nucleobase – nucleoside and nucleotide metabolic process	0.0246	0.0811
Generation of precursor metabolites and energy	0.0060	0.0559	Cofactor biosynthetic process	0.0288	0.0932
Hsa00190: Oxidative phosphorylation	0.0061	0.0214	Oxidoreductase activity – acting on CH-OH group of donors	0.0288	0.0734
Fatty acid metabolic process	0.0064	0.0378	Hsa00650: Butanoate metabolism	0.0295	0.0919
Hsa00640: Propanoate metabolism	0.0084	0.0499	Hsa00280: Valine, leucine, and isoleucine degradation	0.0343	0.0602

* *P*<0.05;

FDR q<0.1.

To unravel whether the observed changes in gene expression are dependent on the high-fat content of the diet or on the type of PUFAs, we also directly compared the expression profiles induced by the two high-fat diets, CO and FO. This comparison resulted in 305 differentially expressed genes. We found enrichment for 124 biological processes (FDR<0.001), of which many were involved in immune response, inflammation, energy metabolism, and cell death pathways (Supplemental Table S2). GSEA **(** *P*<0.05; FDR<0.10) revealed 11 pathways to be down-regulated in the FO group compared with the CO group (Table 6). Of these, 7 pathways were also identified comparing FO versus control. Remarkably, one of the 4 pathways that were found to be down-regulated comparing the two high-fat diet groups, was the insulin receptor signaling pathway. Expression of several genes involved in this pathway, such the insulin receptor (*Inrs*), insulin receptor substrate 1 (*Irs1*), and the p85 regulatory subunit of phosphatidylinositol 3-kinase (*Pik3r1)*, was reduced, although not significantly.

**Table 6:** GSEA pathways down-regulated in the FO group, as compared to CO diet.

Pathway	Nominal*P*-value*	FDRq value
Mitochondrial respiratory chain	0.0000	0.0368
Response to light stimulus†	0.0020	0.0913
Microbody	0.0020	0.0680
Peroxisome	0.0020	0.0544
Insulin receptor signaling pathway†	0.0038	0.0985
Mitochondrial membrane part	0.0039	0.0682
Nuclear body†	0.0041	0.0539
Mitochondrion	0.0059	0.0846
Mitochondrial inner membrane	0.0117	0.0862
Mitochondrial membrane	0.0139	0.0868
Oxidoreductase activity – acting on NADH or NADPH†	0.0151	0.0877

* *P*<0.05; FDR q<0.1.

† Pathways down-regulated in FO samples as compared to CO samples, but not compared to controls.

## Discussion

Using a murine model for early life dietary fat exposure on female pups, the present study found changes in the mammary gland transcriptome that accompanies excessive postnatal weight gain following perinatal exposure to fatty diets high in *n*-6 or in *n*-3 PUFAs. Reproductive parameters such as pregnancy rate, litter size and sex ratio did not differ between the dietary groups. Although not significant, the imbalance in sex ratio in litters born from dams fed a high-fat diet is remarkable. Exposure to high-fat diets has previously been shown to increase the sex ratio in mice, probably through the amount of fat consumed rather than the number of calories consumed [42], [43]. In our study, there may be a real effect, which may be not statistically significant due to the small number of dams in each diet group. FO contains approximately 15% linoleic (LA) and 56% α-linolenic acid (ALA), or an *n*-6 to *n*-3 ratio of approximately 1∶4 whereas CO contains approximately 57% LA and 1% ALA, or an *n*-6 to *n*-3 ratio of 57∶1. The FO test diet specifically, having a low *n*-6 to *n*-3 ratio, affected the serum fatty acid profile of mouse pups. This effect persisted for at least 4 weeks after switching to a normal diet, since serum levels of several *n-*3 PUFAs remained elevated, and serum arachidonic acid was reduced, compared to mice exposed to the control or CO diets. Serum levels of LA were comparable between the groups most likely due to the shift to low-fat control diet from 6 weeks of age onwards. These results indicate that perinatal and early life exposure to the FO diet modulated the serum fatty acid profile by altering circulating long chain PUFAs, which appears to be a more lasting effect. Additional studies will be needed to ascertain how long the altered serum fatty acid profiles last beyond the 10 week postnatal interval evaluated in this murine study.

Postnatal body weight gain of the female offspring was increased after perinatal exposure to a high-fat diet as compared to regular low-fat diet exposure. This increase in body weight persisted until termination of the animals at 10 weeks of age (i.e. 4 weeks after cessation of treatment) when the high-fat diet was based on FO, but not when based on CO. Although additional studies are needed to elucidate whether this is a temporary or permanent effect, we considered this finding rather unexpected, because *n*-6 PUFAs have long been labeled ‘bad’ due to adverse effects on cardiovascular and other health outcomes while *n-*3 PUFAs are generally regarded as ‘protective’ fatty acids. In several studies *n*-3 PUFAs have been demonstrated to prevent obesity and glucose intolerance [44]–[46]. For example, treatment with a high-fat (35%) CO diet supplemented with docosahexaenoic acid derivatives was reported to prevent and reverse obesity in mice [47]. In all these studies, however, treatment occurred postnatally. Exposure to *n-*3 PUFAs occurred in our study during critical developmental periods, when programming of the various tissues and organs was still ongoing. This might explain the (temporary) difference in effect on body weight gain.

Transcriptome analysis of the mammary gland revealed changes in genome-wide gene expression accompanying the effects on serum fatty acid profile and body weight trajectories of the female pups. Stage of estrus has been shown to affect the expression of specific genes, including Wnt-4, Wnt-6, RANKL [48] and TIMP-3, TIMP-4, MMP-9, MMP-13 [49]. An examination of these genes in the present dataset showed a degree of sample to sample variation that could in fact be attributed to collection at different stages of the estrus cycle; however, with exception of TIMP-4, this degree of variation did not correlate with dietary group (data not shown). This differs from Fata et al. [49] who reported co-regulation of TIMP-4 with TIMP-3, MMP-9 and MMP-13 during the mouse estrus cycle and suggests that alterations linked to developmental dietary exposure were stronger and more widely distributed across the genome than those previously linked to the estrus cycle. This is further supported by the consistent pattern of strong differences in gene expression between experimental groups that masks any observable effect of estrus cycle stage. Both test diets, FO and CO, resulted in altered gene expression profiles compared to control low-fat diets. The response induced by the CO diet was weak in comparison to the effect induced by the FO diet, and the few changes in genes observed in the CO diet could not be reliably mapped to a coherent biological theme. On the other hand, the stronger effect of the FO test diet resulted in the identification of several responsive biological processes, mainly down- and up-regulation of pathways associated with energy metabolism and inflammation, respectively. This is not surprising, considering the mammary gland mainly consists of adipose tissue. Adiposity of the target tissue is likely important either as a direct (cellular mass) or indirect (signaling) factor. Adipose tissue has a high capacity to produce and secrete several factors collectively called ‘adipokines’ involved in numerous functions including metabolism, insulin secretion, reproduction, immunity and inflammation [50], [51]. Obesity is recognized as being associated with a chronic, low-grade, inflammation, with altered levels of several circulating factors such as C-reactive protein (CRP), tumor necrosis factor α (TNF-α), and interleukin-6 (IL-6) [50], [52], [53]. Recently, activation of toll-like receptor (TLR) signaling has been recognized as an alternative activator of obesity-induced inflammation [54]. This inflammation is now widely believed to be the key link between obesity and development of insulin resistance and metabolic syndrome [55].

In the present study, mice fed a high-fat diet were shifted to a regular low-fat diet from 6 weeks of age onwards. This relative caloric restriction resulted in a slightly reduced body weight gain in both groups. The observed up-regulation of several inflammation-related pathways in the FO-fed mice is not consistent with findings from studies involving human obese subjects. Those studies showed that in obese subjects, diet-induced weight loss is accompanied by an improvement of the inflammatory profile through a decrease of pro-inflammatory markers and an increase of anti-inflammatory markers [56]–[59]. In juvenile mice, however, more consistent findings have been reported. Diet restriction for two months after a four-month-period of a high-fat diet only partially improved markers of systemic and adipose tissue inflammation [60]. In contrast to the FO test diet, female pups exposed to CO test diet had gene expression profiles only slightly altered versus controls, and without evident regulation of pathways involved in inflammation and energy metabolism. FO and CO test diets, despite being isocaloric, had different effects on postnatal weight gain with the CO-fed mice approaching control weights by the time the experiment was terminated at 10 weeks of age. Consequently, the extent of inflammation in CO-fed mice, if induced at all, might have been less severe or more reversible compared to FO-fed mice. The effects associated with the high-fat test diet based on CO were therefore far less pronounced compared to the FO test diet with respect to gene expression in the mammary gland, ratio of *n*-6 to *n*-3 PUFAs in the serum, and postnatal weight gain.

Additional studies will be needed to determine how these disparate changes are causally linked; however, several interesting connections arise from the annotation of differentially expressed genes. Remarkably, one pathway specifically identified in comparing animals from FO and CO test diets was the insulin signaling pathway. In obesity and type 2 diabetes, insulin resistance is manifested by decreased insulin-stimulated glucose transport and metabolism in adipocytes and one mechanism is impaired insulin signaling [61], [17]. In adipocytes from obese humans with type 2 diabetes, insulin receptor substrate-1 (IRS-1) expression is reduced, resulting in decreased IRS-1–associated phosphoinositide-3 kinase (PI3K) activity [62]. Confirmation that FO-fed mice experience insulin resistance requires a glucose tolerance test. The fact that the insulin pathway was only identified comparing FO- and CO-fed mice, and not when comparing FO-fed mice versus controls, might indicate that the effect was rather weak. Changes in genome-level expression profiles, measured here for the 4th right abdominal mammary gland of 10-week old pups, could also reflect subtle cellular defects or alterations in cellular subpopulations as a consequence of prenatal-lactational maternal dietary conditions. We may speculate that such changes could be precursors to key events affecting the risk of mammary tumors later in life.

The present study did not investigate detailed histopathology of the mammary gland, nor assess the fatty acid composition of the tissues. With regards to the former, detailed histopathology of the mammary gland might help determine whether there were any apparent changes in development or maturation that coincided with the altered gene expression profiles; however, this would best be done at stages beyond when the RNA samples were collected for microarray analysis in the present design. With regards to the latter, we monitored fatty acid composition in the sera and assume this to reflect the fatty acid composition of the mammary tissue rather well, since the serum was collected 4 weeks after the animals shifted from test diet to normal diet. However, perinatal exposure to a high-fat diet based on FO did not affect birth weight, but resulted in an increased body weight gain at 10 weeks of age. Together with an altered gene expression profile of the mammary gland this suggests a lasting effect by diet. Besides increasing the risk of developing obesity, insulin resistance, and ultimately, metabolic syndrome, these effects may also result in altered breast cancer risk by altering development of the mammary gland. Fetal exposure to high-fat diets based on *n*-6 PUFAs has been shown to increase the number of terminal end buds (TEBs) in the mammary gland, while (pre)pubertal exposure to a high-fat diet based on *n*-3 PUFAs or *n*-6 PUFAs has been shown to reduce the number of TEBs [35], [63]–[65]. TEBs are the structures where carcinogen-induced mammary tumors in rats and mice are initiated. The number of TEBs generally, but not always, correlates with breast cancer risk [66], [67]. Further studies on how and at what time point in early life (e.g., during fetal or (pre)pubertal life) dietary exposure to *n*-3 PUFAs alters mammary gland development and subsequent breast cancer risk are warranted.

A considerable number of animal studies suggest that dietary intake of *n-*3 PUFAs may modify breast cancer risk and provide protection against mammary tumorigenesis. In humans, however, evidence of a beneficial relationship between *n-*3 PUFAs and incidence rates of breast cancer from epidemiological studies has been inconclusive (reviewed by MacLennan & Ma [23]). Additional studies are required to demonstrate whether a high-fat diet rich in *n*-3 PUFAs can indeed also be ‘bad’, and not only ‘protective’, and to investigate whether specific pathways can be linked to specific health outcomes such as breast cancer. The protective effect of *n-*3 PUFAs might be dependent on the fat content of the diet. In a previous study by Hilakivi-Clarke and co-workers [64], prepubertal (PND 5– PND 25) dietary exposure of rats to a high-fat *n*-3 PUFA diet has been shown to have adverse effects on the mammary gland, by increasing cell proliferation and reducing the rate of apoptosis. These changes were associated with a significantly increased risk of carcinogen-induced mammary tumorigenesis. Subsequent transcriptome analyses of the mammary gland after a 3–4 week period of control diet resulted in gene expression profiles significantly different from those induced by a *n*-6 diet [67]. Although the exposure regimen in that study was prepubertal and not perinatal, we consider these results supportive of our findings that maternal diet indeed has a lasting effect on the mammary gland. Further studies are needed to localize the susceptible stage(s). Assuming the current findings in mice reflect the human response, then the present study suggests the nature and degree of dietary fat consumption during fetal and/or lactational development may contribute to the susceptibility to breast cancer risk later in life.

## Materials and Methods

### Ethics Statement

The animal protocol used in this research was reviewed and approved by the institute’s ethical committee on experimental animals, in accordance to national legislation for RIVM (the performance site for the animal work) and the Institutional Animal Care and Use Committee of the University of Louisville (the host institution for the bioinformatics part of this study). Carbon dioxide asphyxiation was the method of euthanasia. This study was agreed upon by the Animal Experimentation Ethical Committee of the RIVM under permit number 200500400. Animal handling in this study was carried out in accordance with relevant Dutch national legislation, including the 1997 Dutch Act on Animal Experimentation.

### Mice and Treatment Diets

Wild-type FVB/NHanHsd mice, six to eight-weeks old, were obtained from Harlan (Horst, NL). All mice were housed in an animal facility at RIVM in a climate-controlled room with a 12 h on/off light cycle. Tap water and diets as described below were provided *ad libitum*. Animals were monitored daily for general health. All diets used in this experiment were obtained from AB Diets (Woerden, NL). Mice in the control group were fed a low-fat (5% fat) regular diet. The high-fat diets contained 24% fat at the expense of carbohydrates and were based on corn oil (CO; high ratio of *n*-6/*n*-3 PUFAs) or flaxseed oil (FO; low ratio of *n*-6/*n*-3 PUFAs) (Chempri Oleochemicals, Raamsdonksveer, NL). Compositions of the pelleted diets are shown in Table 7. Similar diets have been previously used in our group [68].

**Table 7:** Composition of treatment diets.

Component*	Low-fat(control)	High-fat(corn oil)	High-fat (flaxseed oil)
Corn Starch	100	186.7	186.7
Cerelose/dextrose	543	203.8	203.8
Casein protein	200	240	240
Corn oil	50	240	–
Flaxseed oil	–	–	240
Vitamin premix	2.5	3	3
Mineral premix	2.5	3	3
Cellulose/Didacel 2+4	50	60	60
Methionine (synthetic)	2	3	3
Other	50	60.5	60.5
Total	1000	1000	1000
Gross Energy (kcal/g)	3.8	4.7	4.7

* Components are indicated in g per 1000 grams.

### Experimental Design

A schematic overview of the experimental study design is given in Figure 1. The study consisted of two dietary exposure groups and one control group, with differing proportions of PUFAs in the diet. Each dietary group consisted of 6 females and 3 males. After one week of acclimation to the animal room the parental dams and sires were shifted from standard mouse chow to one of the treatment diets shown in Table 7 for two-weeks prior to mating. The females and males were then allowed to breed with one another during a one week period. Subsequently, impregnated dams were housed individually and maintained on their respective experimental diets through pregnancy and lactation. The number of live-born pups was recorded at parturition, designated postnatal day (PND) 0. Body weights of all pups were recorded on PND 1, PND 7, and PND 14. F1 generation female pups were weaned on PND 21 (male pups and dams were terminated). Post-weaned F1 females were then kept on the respective treatment diets until 6 weeks of age and then shifted to a standard low-fat (5% fat) chow until 10 weeks of age, when the animals were terminated. This experimental design led to the F1 females being exposed to test diets during three serial life stages: (1) parentally from at least two-weeks prior to conception; (2) maternally through gestation and lactation; and (3) post-weaning through 6-weeks of age. The four-week recovery interval between 6- and 10 weeks on normal chow followed the hypothesis that early lifestage diet would have a lasting impression on the transcriptome of the adult mammary gland.

Body weights of F1 female pups (*n* = 21 born in 5 litters for the control group, *n* = 12 born in 4 litters for the CO diet group, and *n* = 15 born in 5 litters for the FO diet group) were recorded weekly from PND 21 until 10 weeks of age. At the termination of the protocol, the mice were euthanized and blood and major organs isolated from each. The right fourth abdominal mammary gland was excised and kept in RNA*later* RNA stabilization Solution (Ambion, Austin, Texas) at 4°C. The liver, kidney and spleen were snap-frozen in liquid nitrogen for future reference.

### RNA Isolation, Microarray Hybridization and Gene Expression Analysis

Total RNA from tissue samples was isolated. DNA/RNA was extracted by using AllPrep DNA/RNA mini isolation kit (Qiagen, Valencia, CA, USA). RNA samples were treated with the RNase-Free DNase set (Qiagen) and RNA was assessed for quality with the Bioanalyzer 2100 (Agilent Technologies, Palo Alto, CA, USA). High-quality RNA was labeled and hybridized to spotted 65-mer oligonucleotide microarrays. Technical details about the mouse 65-mer oligonucleotide library, printing, quality checks, labeling and hybridization can be found in Bruins *et al*. [69], [70]. The libraries represent in total 21,766 LEADS™ clusters plus 231 controls. Total RNA samples, labeled with Cy3, were hybridized in randomized batches, according to a common reference design without dye swap, with a RNA pool of all samples isolated serving as common reference (Cy5). RNA amplification and labeling were carried out with Amino Allyl MessageAmp aRNA Kit (Ambion, Austin, Texas, USA), using 1 µg of total RNA as starting material.

### Pre-processing of Microarray Dataset

The resulting datasets were submitted to the ArrayExpress (http://www.ebi.ac.uk/arrayexpress) repository with the title “Mouse mammary gland developmental programming by dietary exposure” (E-TABM-1185) and data storage was in compliance with MIAME guidelines. The normalized data consisted of 28 arrays, representing 3 batch groups: Group A (*n = *12) was from the low-fat (5%) control group, and Groups B and C (*n = *8 each) were from the high-fat treatment groups. Microarray quality control was performed on raw data by means of visual inspection of the scanned images, as well as a check on the scatter and MA plots that compares differences in the log2-intensity ratio (M) as a function of the average intensity for a dot in the plot (A) to assess the intensity-dependent ratio of raw microarray data. The BioConductor software packages *arrayQuality* and *marray* from www.bioconductor.org were used to assess array quality. Bioconductor is an open source and open development project based on the R programming language-providing tools for the analysis of high-throughput genomic data. An offset of 50 was used for background correction to avoid negative or zero corrected intensities and to preserve variance for analysis of differential expression. Locally weighted regression was used to normalize within arrays and quantile normalization was performed across arrays because of the large variation between arrays seen in the box plots.

### Microarray Data Analysis

To identify genes that were differentially expressed between the treatment groups and the control group, ratiometric values (test/reference) were transformed to log_2_ and normalized with Lowess smoothing. A ‘source of variance’ plot mapped each sample and characterized the systemic variance for the dams, dates of hybridization of the arrays, and treatments. Batch and litter-size effects were removed using Partek Genomics Suite v 6.3 (Partek Inc.; www.partek.com) and the residual variance was analyzed by Principal Components Analysis (PCA), one-way ANOVA (*P*≤0.005) and specific group comparisons. All data was mean-centered and quantile-normalized to normalize gene expression distributions across the different microarrays.

Overrepresented biological processes, based on Gene Ontology (GO) terms, in the various sets of differentially expressed genes were identified using MetaCore (GeneGo Inc, St. Joseph, MI), which is an integrated manually-curated database and software suite for pathway analysis (http://www.genego.com/metacore.php). Groupwise regulation of Gene Ontology categories and KEGG pathways was determined by Gene Set Enrichment Analysis (GSEA) [71] using default analysis parameters and 1,000 permutations. Gene set collections used were the c5.all (Gene Ontology) and c2.kegg gene sets provide by MsigDB (http://www.broad.mit.edu/gsea/msigdb/). Gene sets were considered regulated if the GSEA *P* -value was <0.05 and the False Discovery Rate (FDR) was <0.10.

### Real-time Quantitative RT-PCR (qRT-PCR)

Expression levels of several genes were measured in all samples by means of real-time quantitative PCR. All reagents and equipment were obtained from Applied Biosystems (Foster City, USA). The following TaqMan® Gene Expression Assays were used: Mm00441259_g1 (*Ccl3*), Mm00438297_g1 (*Cox7a1*), Mm00432648_m1 (*Cox8b*), Mm00445259_m1 (*Il4*), and Mm01333032_m1 (*Syk*). The assay for the *Hprt* gene was custom made and included as endogenous control. Primer and probe sequences were as follows: *Hprt*_forward: 5′-GCT GAC CTG CTG GAT TAC ATT AAA-3′; *Hprt*_reverse: 5′-TTG ACT GGT CAT TAC AGT AGC TCT TCA-3′; *Hprt*_probe: 5′-CGC TGA ATA GAA ATA GTG-3′.

Presence of genomic DNA in RNA samples and amplification efficiency for all assays were assessed prior to the measurements. RNA was converted to cDNA using the High-Capacity cDNA Archive Kit according to the manufacturer’s instructions. For each measured gene, 1 µL of TaqMan® Gene Expression Assay was mixed with 10 µL TaqMan® Fast Universal PCR Master mix and added to 50 ng of every cDNA sample in 9 µL Milli-Q in duplicate. The cDNA was amplified in a 96-well plate during 40 cycles of 3 s at 95°C and 30 s at 60°C, preceded by 20 s at 95°C for enzyme activation, using the 7500 Fast Real-Time PCR System. No template controls (NTC) were included in all plates. Threshold cycles (CT) were automatically derived from the amplification plots constructed of the ROX-normalized fluorescence signals by 7500 Fast System SDS Software v2.0.6. The mean of the *Hprt* gene expression level of all samples was used to normalize the expression of the other genes. Relative quantification of the mRNA copies in all samples compared to controls was performed by the comparative CT method (ΔΔCt) using Microsoft Excel.

### Fatty Acid Profiling of Mouse Sera

Fatty acid composition was analyzed in a representative number of sera obtained from mice fed these diets at terminal section (n = 8−10). Methods were adapted from procedures described earlier [72]. Fatty acids have been expressed as percent of the total fatty acids present in the chromatogram and are reported as the mean and standard deviation (SD) for each treatment group.

### Statistical Analyses

General statistical procedures were performed with either SPSS version 12.0.1 or S-PLUS 2000 statistical software packages. All values were expressed as means ± standard deviations when appropriate. Reproductive parameters, fatty acid serum levels, and pup weights of PND 1 were analyzed using one-way ANOVA followed by either a Bonferroni’s or Dunnett’s multiple comparison tests for post hoc analysis. Body weights of female pups from 3 to 10 weeks of age were analyzed using a nonlinear repeated measures model. The nonlinearity is formed by an asymptotic growth model, in which the weight y_ij_ of mouse i at time t_j_ is modeled as follows:
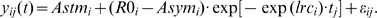



## Supporting Information

Table S1 **List of 670 genes that classified samples by treatment.**
(XLS)

Table S2 **Biological processes over-represented in genes differentially expressed in FO versus CO samples.**
(PDF)

## References

[1] Eriksson JG, Forsen T, Tuomilehto J, Jaddoe VW, Osmond C, et al (2002) Effects of size at birth and childhood growth on the insulin resistance syndrome in elderly individuals. Diabetologia 45: 342–8. 11914739 10.1007/s00125-001-0757-6

[2] Fall CH, Pandit AN, Law CM, Yajnik CS, Clark PM, et al (1995) Size at birth and plasma insulin-like growth factor-1 concentrations. Arch Dis Child 73: 287–93. 7492190 10.1136/adc.73.4.287 PMC1511321

[3] Hales CN, Barker DJ, Clark PM, Cox LJ, Fall C, et al (1991) Fetal and infant growth and impaired glucose tolerance at age 64. BMJ 303: 1019–22. 1954451 10.1136/bmj.303.6809.1019 PMC1671766

[4] Yajnik CS, Fall CH, Vaidya U, Pandit AN, Bavdekar A, et al (1995) Fetal growth and glucose and insulin metabolism in four-year-old Indian children. Diabet Med 12: 330–6. 7600749 10.1111/j.1464-5491.1995.tb00487.x

[5] Armitage JA, Khan IY, Taylor PD, Nathanielsz PW, Poston L (2004) Developmental Programming of Metabolic Syndrome by Maternal Nutritional Imbalance; How Strong is the Evidence from Experimental Models in Mammals? J Physiol 561: 355–77. 15459241 10.1113/jphysiol.2004.072009 PMC1665360

[6] Bertram CE, Hanson MA (2001) Animal models and programming of the metabolic syndrome. Br Med Bull 60: 103–21. 11809621 10.1093/bmb/60.1.103

[7] Barker DJ, Osmond C (1986) Infant mortality, childhood nutrition, and ischaemic heart disease in England and Wales. Lancet 1: 1077–81. 2871345 10.1016/s0140-6736(86)91340-1

[8] Barker DJ, Osmond C, Golding J, Kuh D, Wadsworth ME (1989) Growth in utero, blood pressure in childhood and adult life, and mortality from cardiovascular disease. BMJ 298: 564–7. 2495113 10.1136/bmj.298.6673.564 PMC1835925

[9] Barker DJ (2004) The developmental origins of adult disease. J Am Coll Nutr 23: 588S–95S. 15640511 10.1080/07315724.2004.10719428

[10] Barker DJ, Bagby SP, Hanson MA (2006) Mechanisms of disease: in utero programming in the pathogenesis of hypertension. Nat Clin Pract Nephrol 2: 700–7. 17124527 10.1038/ncpneph0344

[11] Bouret SG (2009) Early life origins of obesity: role of hypothalamic programming. J Pediatr Gastroenterol Nutr 48 Suppl 1S31–S38. 19214056 10.1097/MPG.0b013e3181977375

[12] Gardner DS, Bell RC, Symonds ME (2007) Fetal mechanisms that lead to later hypertension. Curr Drug Targets 8: 894–905. 17691926 10.2174/138945007781386901

[13] Martin-Gronert MS, Ozanne SE (2005) Programming of appetite and type 2 diabetes. Early Hum Dev 81: 981–8. 16257499 10.1016/j.earlhumdev.2005.10.006

[14] Cottrell EC, Ozanne SE (2008) Early life programming of obesity and metabolic disease. Physiol Behav 94: 17–28. 18155097 10.1016/j.physbeh.2007.11.017

[15] Muhlhausler BS (2007) Programming of the appetite-regulating neural network: a link between maternal overnutrition and the programming of obesity? J Neuroendocrinol 19: 67–72. 17184487 10.1111/j.1365-2826.2006.01505.x

[16] Reusens B, Ozanne SE, Remacle C (2007) Fetal determinants of type 2 diabetes. Curr Drug Targets 8: 935–41. 17691930 10.2174/138945007781386866

[17] Samuelsson AM, Matthews PA, Argenton M, Christie MR, McConnell JM, et al (2008) Diet-induced obesity in female mice leads to offspring hyperphagia, adiposity, hypertension, and insulin resistance: a novel murine model of developmental programming. Hypertension 51: 383–92. 18086952 10.1161/HYPERTENSIONAHA.107.101477

[18] Hortobagyi GN, de la Garza SJ, Pritchard K, Amadori D, Haidinger R, et al (2005) The global breast cancer burden: variations in epidemiology and survival. Clin Breast Cancer 6: 391–401. 16381622 10.3816/cbc.2005.n.043

[19] Parkin DM, Bray F, Ferlay J, Pisani P (2005) Global cancer statistics, 2002. CA Cancer J Clin 55: 74–108. 15761078 10.3322/canjclin.55.2.74

[20] Ziegler RG, Hoover RN, Pike MC, Hildesheim A, Nomura AM, et al (1993) Migration patterns and breast cancer risk in Asian-American women. J Natl Cancer Inst 85: 1819–27. 8230262 10.1093/jnci/85.22.1819

[21] Fay MP, Freedman LS, Clifford CK, Midthune DN (1997) Effect of different types and amounts of fat on the development of mammary tumors in rodents: a review. Cancer Res 57: 3979–88. 9307282

[22] Khalid S, Hwang D, Babichev Y, Kolli R, Altamentova S, et al (2010) Evidence for a tumor promoting effect of high-fat diet independent of insulin resistance in HER2/Neu mammary carcinogenesis. Breast Cancer Res Treat 122: 647–59. 19851863 10.1007/s10549-009-0586-8

[23] Maclennan M, Ma DW (2010) Role of dietary fatty acids in mammary gland development and breast cancer. Breast Cancer Res 12: 211. 21067551 10.1186/bcr2646 PMC3096965

[24] Moral R, Escrich R, Solanas M, Vela E, Costa I, et al (2011) Diets high in corn oil or extra-virgin olive oil provided from weaning advance sexual maturation and differentially modify susceptibility to mammary carcinogenesis in female rats. Nutr Cancer 63: 410–20. 21391126 10.1080/01635581.2011.535956

[25] Welsch CW (1992) Relationship between dietary fat and experimental mammary tumorigenesis: a review and critique. Cancer Res 52: 2040s–8s. 1544139

[26] Boyd NF, Stone J, Vogt KN, Connelly BS, Martin LJ, et al (2003) Dietary fat and breast cancer risk revisited: a meta-analysis of the published literature. Br J Cancer 89: 1672–85. 14583769 10.1038/sj.bjc.6601314 PMC2394401

[27] Howe GR, Hirohata T, Hislop TG, Iscovich JM, Yuan JM, et al (1990) Dietary factors and risk of breast cancer: combined analysis of 12 case-control studies. J Natl Cancer Inst 82: 561–9. 2156081 10.1093/jnci/82.7.561

[28] Sieri S, Krogh V, Ferrari P, Berrino F, Pala V, et al (2008) Dietary fat and breast cancer risk in the European Prospective Investigation into Cancer and Nutrition. Am J Clin Nutr 88: 1304–12. 18996867 10.3945/ajcn.2008.26090

[29] Thiebaut AC, Kipnis V, Chang SC, Subar AF, Thompson FE, et al (2007) Dietary fat and postmenopausal invasive breast cancer in the National Institutes of Health-AARP Diet and Health Study cohort. J Natl Cancer Inst 99: 451–62. 17374835 10.1093/jnci/djk094

[30] Burdge GC, Lillycrop KA, Jackson AA (2009) Nutrition in early life, and risk of cancer and metabolic disease: alternative endings in an epigenetic tale? Br J Nutr 101: 619–30. 19079817 10.1017/S0007114508145883 PMC2649281

[31] Michels KB, Xue F (2006) Role of birthweight in the etiology of breast cancer. Int J Cancer 119: 2007–25. 16823839 10.1002/ijc.22004

[32] Xue F, Michels KB (2007) Intrauterine factors and risk of breast cancer: a systematic review and meta-analysis of current evidence. Lancet Oncol 8: 1088–100. 18054879 10.1016/S1470-2045(07)70377-7

[33] McMillen IC, Robinson JS (2005) Developmental origins of the metabolic syndrome: prediction, plasticity, and programming. Physiol Rev 85: 571–633. 15788706 10.1152/physrev.00053.2003

[34] De Assis S, Hilakivi-Clarke L (2006) Timing of dietary estrogenic exposures and breast cancer risk. Ann N Y Acad Sci 1089: 14–35. 17261753 10.1196/annals.1386.039

[35] Hilakivi-Clarke L, Stoica A, Raygada M, Martin MB (1998) Consumption of a high-fat diet alters estrogen receptor content, protein kinase C activity, and mammary gland morphology in virgin and pregnant mice and female offspring. Cancer Res 58: 654–60. 9485017

[36] Hilakivi-Clarke L, Cho E, Cabanes A, deAssis S, Olivo S, et al (2002) Dietary Modulation of Pregnancy Estrogen Levels and Breast Cancer Risk among Female Rat Offspring. Clin Cancer Res 8: 3601–10. 12429652

[37] Hilakivi-Clarke L, Clarke R, Lippman M (1999) The influence of maternal diet on breast cancer risk among female offspring. Nutrition 15: 392–401. 10355854 10.1016/s0899-9007(99)00029-5

[38] Hilakivi-Clarke L, Onojafe I, Raygada M, Cho E, Skaar T, et al (1999) Prepubertal exposure to zearalenone or genistein reduces mammary tumorigenesis. Br J Cancer 80: 1682–8. 10468283 10.1038/sj.bjc.6690584 PMC2363126

[39] Ion G, Akinsete JA, Hardman WE (2010) Maternal consumption of canola oil suppressed mammary gland tumorigenesis in C3(1) TAg mice offspring. BMC Cancer 10: 81. 20205934 10.1186/1471-2407-10-81 PMC2846884

[40] Kavanagh K, Sajadian S, Jenkins KA, Wilson MD, Carr JJ, et al (2010) Neonatal and fetal exposure to trans-fatty acids retards early growth and adiposity while adversely affecting glucose in mice. Nutr Res 30: 418–26. 20650350 10.1016/j.nutres.2010.06.006 PMC2910901

[41] Shankar K, Kang P, Harrell A, Zhong Y, Marecki JC, et al (2010) Maternal overweight programs insulin and adiponectin signaling in the offspring. Endocrinology 151: 2577–89. 20371699 10.1210/en.2010-0017 PMC2875830

[42] Rosenfeld CS, Grimm KM, Livingston KA, Brokman AM, Lamberson WE, et al (2003) Striking variation in the sex ratio of pups born to mice according to whether maternal diet is high in fat or carbohydrate. Proc Natl Acad Sci U S A 100: 4628–32. 12672968 10.1073/pnas.0330808100 PMC153606

[43] Alexenko AP, Mao J, Ellersieck MR, Davis AM, Whyte JJ, et al (2007) The contrasting effects of ad libitum and restricted feeding of a diet very high in saturated fats on sex ratio and metabolic hormones in mice. Biol Reprod 77: 599–604. 17522073 10.1095/biolreprod.107.062174

[44] Fedor D, Kelley DS (2009) Prevention of insulin resistance by n-3 polyunsaturated fatty acids. Curr Opin Clin Nutr Metab Care 12: 138–46. 19202385 10.1097/MCO.0b013e3283218299

[45] Kopecky J, Rossmeisl M, Flachs P, Kuda O, Brauner P, et al (2009) n-3 PUFA: bioavailability and modulation of adipose tissue function. Proc Nutr Soc 68: 361–9. 19698199 10.1017/S0029665109990231

[46] White PJ, Arita M, Taguchi R, Kang JX, Marette A (2010) Transgenic restoration of long chain {omega}-3 fatty acids in insulin target tissues improves resolution capacity and alleviates obesity-linked inflammation and insulin resistance in high fat-fed mice. Diabetes. 10.2337/db10-0054 PMC2992767 20841610

[47] Rossmeisl M, Jelenik T, Jilkova Z, Slamova K, Kus V, et al (2009) Prevention and reversal of obesity and glucose intolerance in mice by DHA derivatives. Obesity (Silver Spring) 17: 1023–31. 19148125 10.1038/oby.2008.602

[48] Silberstein GB, Van Horn K, Hrabeta-Robinson E, Compton J (2006) Estrogen-triggered delays in mammary gland gene expression during the estrous cycle: evidence for a novel timing system. J Endocrinol 190: 225–39. 16899557 10.1677/joe.1.06725

[49] Fata JE, Chaudhary V, Khokha R (2001) Cellular turnover in the mammary gland is correlated with systemic levels of progesterone and not 17beta-estradiol during the estrous cycle. Biol Reprod 65: 680–8. 11514328 10.1095/biolreprod65.3.680

[50] Das UN (2002) Obesity, metabolic syndrome X, and inflammation. Nutrition 18: 430–2. 11985951 10.1016/s0899-9007(01)00747-x

[51] Mora S, Pessin JE (2002) An adipocentric view of signaling and intracellular trafficking. Diabetes Metab Res Rev 18: 345–56. 12397577 10.1002/dmrr.321

[52] Engstrom G, Hedblad B, Stavenow L, Lind P, Janzon L, et al (2003) Inflammation-sensitive plasma proteins are associated with future weight gain. Diabetes 52: 2097–101. 12882928 10.2337/diabetes.52.8.2097

[53] Grimble RF (2002) Inflammatory status and insulin resistance. Curr Opin Clin Nutr Metab Care 5: 551–9. 12172480 10.1097/00075197-200209000-00015

[54] Konner AC, Bruning JC (2011) Toll-like receptors: linking inflammation to metabolism. Trends Endocrinol Metab 22: 16–23. 20888253 10.1016/j.tem.2010.08.007

[55] de Luca C, Olefsky JM (2008) Inflammation and insulin resistance. FEBS Lett 582: 97–105. 18053812 10.1016/j.febslet.2007.11.057 PMC2246086

[56] Clement K, Viguerie N, Poitou C, Carette C, Pelloux V, et al (2004) Weight loss regulates inflammation-related genes in white adipose tissue of obese subjects. FASEB J 18: 1657–69. 15522911 10.1096/fj.04-2204com

[57] Cottam DR, Mattar SG, Barinas-Mitchell E, Eid G, Kuller L, et al (2004) The chronic inflammatory hypothesis for the morbidity associated with morbid obesity: implications and effects of weight loss. Obes Surg 14: 589–600. 15186624 10.1381/096089204323093345

[58] Esposito K, Pontillo A, Di Palo C, Giugliano G, Masella M, et al (2003) Effect of weight loss and lifestyle changes on vascular inflammatory markers in obese women: a randomized trial. JAMA 289: 1799–804. 12684358 10.1001/jama.289.14.1799

[59] van Dielen FM, Buurman WA, Hadfoune M, Nijhuis J, Greve JW (2004) Macrophage inhibitory factor, plasminogen activator inhibitor-1, other acute phase proteins, and inflammatory mediators normalize as a result of weight loss in morbidly obese subjects treated with gastric restrictive surgery. J Clin Endocrinol Metab 89: 4062–8. 15292349 10.1210/jc.2003-032125

[60] Kalupahana NS, Voy BH, Saxton AM, Moustaid-Moussa N (2010) Energy-Restricted High-Fat Diets Only Partially Improve Markers of Systemic and Adipose Tissue Inflammation. Obesity 19: 245–54. 20847734 10.1038/oby.2010.196

[61] Kahn BB, Flier JS (2000) Obesity and insulin resistance. J Clin Invest 106: 473–81. 10953022 10.1172/JCI10842 PMC380258

[62] Rondinone CM, Wang LM, Lonnroth P, Wesslau C, Pierce JH, et al (1997) Insulin receptor substrate (IRS) 1 is reduced and IRS-2 is the main docking protein for phosphatidylinositol 3-kinase in adipocytes from subjects with non-insulin-dependent diabetes mellitus. Proc Natl Acad Sci U S A 94: 4171–5. 9108124 10.1073/pnas.94.8.4171 PMC20591

[63] Hilakivi-Clarke L, Clarke R, Onojafe I, Raygada M, Cho E, et al (1997) A maternal diet high in n - 6 polyunsaturated fats alters mammary gland development, puberty onset, and breast cancer risk among female rat offspring. Proc Natl Acad Sci U S A 94: 9372–7. 9256489 10.1073/pnas.94.17.9372 PMC23197

[64] Olivo SE, Hilakivi-Clarke L (2005) Opposing effects of prepubertal low- and high-fat n-3 polyunsaturated fatty acid diets on rat mammary tumorigenesis. Carcinogenesis 26: 1563–72. 15888492 10.1093/carcin/bgi118

[65] Olson LK, Tan Y, Zhao Y, Aupperlee MD, Haslam SZ (2010) Pubertal exposure to high fat diet causes mouse strain-dependent alterations in mammary gland development and estrogen responsiveness. Int J Obes (Lond) 34: 1415–26. 20231845 10.1038/ijo.2010.51 PMC2923244

[66] Hilakivi-Clarke L (2007) Nutritional modulation of terminal end buds: its relevance to breast cancer prevention. Curr Cancer Drug Targets 7: 465–74. 17691906 10.2174/156800907781386641

[67] Olivo-Marston SE, Zhu Y, Lee RY, Cabanes A, Khan G, et al (2008) Gene signaling pathways mediating the opposite effects of prepubertal low-fat and high-fat n-3 polyunsaturated fatty acid diets on mammary cancer risk. Cancer Prev Res (Phila) 1: 532–45. 19139003 10.1158/1940-6207.CAPR-08-0030 PMC2673718

[68] Luijten M, Verhoef A, Dormans JA, Beems RB, Cremers HW, et al (2007) Modulation of mammary tumor development in Tg.NK (MMTV/c-neu) mice by dietary fatty acids and life stage-specific exposure to phytoestrogens. Reprod Toxicol 23: 407–13. 17229545 10.1016/j.reprotox.2006.12.001

[69] Bruins W, Bruning O, Jonker MJ, Zwart E, van der Hoeven TV, et al (2008) The absence of Ser389 phosphorylation in p53 affects the basal gene expression level of many p53-dependent genes and alters the biphasic response to UV exposure in mouse embryonic fibroblasts. Mol Cell Biol 28: 1974–87. 18195040 10.1128/MCB.01610-07 PMC2268391

[70] Bruins W, Jonker MJ, Bruning O, Pennings JL, Schaap MM, et al (2007) Delayed expression of apoptotic and cell-cycle control genes in carcinogen-exposed bladders of mice lacking p53.S389 phosphorylation. Carcinogenesis 28: 1814–23. 17317680 10.1093/carcin/bgm041

[71] Subramanian A, Tamayo P, Mootha VK, Mukherjee S, Ebert BL, et al (2005) Gene set enrichment analysis: a knowledge-based approach for interpreting genome-wide expression profiles. Proc Natl Acad Sci U S A 102: 15545–50. 16199517 10.1073/pnas.0506580102 PMC1239896

[72] Mamalakis G, Jansen E, Cremers H, Kiriakakis M, Tsibinos G, et al (2006) Depression and adipose and serum cholesteryl ester polyunsaturated fatty acids in the survivors of the seven countries study population of Crete. Eur J Clin Nutr 60: 1016–1023. 16482070 10.1038/sj.ejcn.1602413

