# Supplementary material for: Lasting Effects on Body Weight and Mammary Gland Gene Expression in Female Mice upon Early Life Exposure to n-3 but Not n-6 High-Fat Diets
Source: PLoS One. 2013 Feb 7;8(2):e55603. doi: 10.1371/journal.pone.0055603 (PMC3567116; doi:10.1371/journal.pone.0055603)
Supplement: Table S2 — Biological processes over-represented in genes differentially expressed in FO versus CO samples. (PDF) [file pone.0055603.s002.pdf]

Table S2. Biological processes over-represented in genes differentially expressed in FO versus CO samples

GO Biological Process	*P*-value (FDR<0.001)	Count
regulation of biological quality	6.496e-13	52/2290
cellular process	1.106e-12	141/12523
homeostatic process	1.422e-12	36/1176
small molecule metabolic process	4.138e-12	46/1930
response to wounding	4.545e-12	30/852
response to stress	1.465e-11	53/2567
generation of precursor metabolites and energy	1.544e-11	21/417
defense response	1.646e-11	31/958
immune system process	3.063e-11	37/1378
inflammatory response	9.065e-11	22/508
cell activation	1.166e-10	21/465
chemical homeostasis	4.502e-10	27/839
leukocyte activation	5.480e-10	19/407
localization	7.808e-10	64/3876
neutrophil chemotaxis	1.408e-9	8/43
locomotory behavior	1.999e-9	18/391
T cell proliferation	2.972e-9	8/47
lipid metabolic process	3.995e-9	29/1061
cellular homeostasis	5.475e-9	24/752
cellular ion homeostasis	5.620e-9	22/634
developmental process	6.483e-9	68/4481
cellular chemical homeostasis	7.672e-9	22/645
lymphocyte activation	8.321e-9	16/331
leukocyte migration	9.457e-9	10/104
locomotion	1.329e-8	22/665
metabolic process	1.383e-8	107/9017
ion homeostasis	1.878e-8	22/678
transport	1.953e-8	55/3316
behavior	2.068e-8	23/743
response to stimulus	2.649e-8	73/5157
establishment of localization	3.105e-8	55/3360
T cell activation	4.048e-8	12/192
activated T cell proliferation	5.328e-8	5/14
leukocyte mediated cytotoxicity	7.015e-8	6/28
lymphocyte proliferation	7.656e-8	6/70
leukocyte chemotaxis	8.571e-8	8/71
oxidative phosphorylation	8.714e-8	10/131
mononuclear cell proliferation	1.069e-7	8/73
leukocyte proliferation	1.190e-7	8/74
ion transmembrane transport	1.836e-7	16/414
carboxylic acid metabolic process	1.921e-7	21/710
oxoacid metabolic process	1.921e-7	21/710
cell chemotaxis	1.993e-7	8/79
organic acid metabolic process	2.311e-7	21/718
taxis	2.402e-7	12/226
chemotaxis	2.402e-7	12/226
cellular ketone metabolic process	2.836e-7	21/727
energy coupled proton transport, down electrochemical gradient	3.095e-7	7/57
ATP synthesis coupled proton transport	3.095e-7	7/57
immune response	3.108e-7	23/864

regulation of transport	4.115e-7	23/878
multicellular organismal process	4.797e-7	78/6072
leukocyte mediated immunity	6.338e-7	10/162
death	6.618e-7	24/973
catabolic process	8.388e-7	30/1441
cation transport	1.090e-6	20/723
multicellular organismal development	1.092e-6	59/4140
monocarboxylic acid metabolic process	1.215e-6	15/420
oxidation reduction	1.228e-6	21/796
anatomical structure development	1.332e-6	55/3758
system development	1.422e-6	52/3467
cellular component movement	1.525e-6	20/739
regulation of intracellular pH	1.978e-6	5/27
cellular monovalent inorganic cation homeostasis	2.007e-6	6/48
cell death	2.071e-6	23/965
regulation of multicellular organismal process	2.104e-6	32/1672
immune response-activating cell surface receptor signaling pathway	2.258e-6	7/76
cell proliferation	2.452e-6	19/695
response to external stimulus	2.592e-6	23/978
regulation of response to stimulus	2.921e-6	21/841
cellular lipid metabolic process	3.625e-6	19/714
immune response-regulating cell surface receptor signaling pathway	3.775e-6	7/82
localization of cell	3.836e-6	16/522
cell motility	3.836e-6	16/522
regulation of response to stress	4.026e-6	16/524
regulation of cellular pH	4.059e-6	5/31
cell migration	4.570e-6	15/468
regulation of apoptosis	4.690e-6	26/1244
alcohol metabolic process	4.703e-6	17/594
regulation of localization	5.061e-6	25/1171
homeostasis of number of cells	5.559e-6	9/162
regulation of programmed cell death	5.730e-6	26/1258
myeloid leukocyte activation	6.059e-6	7/88
cell killing	6.203e-6	6/58
regulation of cell death	7.276e-6	26/1275
organ development	7.290e-6	42/2681
lymphocyte mediated immunity	7.442e-6	8/127
regulation of cytokine production	7.705e-6	12/315
positive regulation of immune response	8.350e-6	11/265
phagocytosis	8.750e-6	7/93
biological regulation	9.335e-6	103/9542
cellular cation homeostasis	9.921e-6	14/438
positive regulation of transport	9.921e-6	14/438
cellular response to chemical stimulus	1.141e-5	19/774
positive regulation of response to stimulus	1.156e-5	14/444
positive regulation of phagocytosis	1.307e-5	5/39
electron transport chain	1.443e-5	8/139
iron ion transport	1.485e-5	5/40
proton transport	1.507e-5	7/101
intracellular pH reduction	1.614e-5	4/20
glycolipid metabolic process	1.680e-5	5/41
immune effector process	1.688e-5	10/234
hydrogen transport	1.714e-5	7/103
response to biotic stimulus	1.789e-5	18/728

cellular extravasation	1.981e-5	4/21
respiratory burst	1.981e-5	4/21
small molecule biosynthetic process	2.037e-5	16/597
immune response-activating signal transduction	2.197e-5	7/107
pH reduction	2.407e-5	4/22
phagocytosis, engulfment	2.407e-5	4/22
response to other organism	2.442e-5	16/606
cation homeostasis	2.501e-5	14/476
monovalent inorganic cation homeostasis	2.559e-5	6/74
response to lipopolysaccharide	2.593e-5	10/246
regulation of phagocytosis	2.671e-5	5/45
ion transport	2.691e-5	21/974
negative regulation of catabolic process	2.982e-5	6/76
monosaccharide metabolic process	3.079e-5	11/305
immune response-regulating signaling pathway	3.703e-5	7/116
response to bacterium	3.885e-5	13/432
ATP biosynthetic process	3.913e-5	7/117
positive regulation of nitric oxide biosynthetic process	4.487e-5	5/50
cellular metabolic process	4.702e-5	87/7860
response to molecule of bacterial origin	4.716e-5	10/264
